# New Therapies of Neovascular AMD beyond Anti-VEGF Injections

**DOI:** 10.3390/vision2010015

**Published:** 2018-03-19

**Authors:** Greggory M. Gahn, Arshad M. Khanani

**Affiliations:** 1 University of Nevada, Reno School of Medicine, Reno, NV 89557, USA; 2 Sierra Eye Associates, Reno, NV 89502, USA

**Keywords:** age-related macular degeneration, Tie2, anti-VEGF, RG7716, ranibizumab

## Abstract

Neovascular age-related macular degeneration is a leading cause of vision loss among the aging population. The current standard of care to treat neovascular age-related macular degeneration is inhibiting vascular endothelial growth factor (VEGF) through intravitreal injections. Recent studies have demonstrated that the tyrosine kinase with immunoglobulin-like and epidermal growth factor-like domains 2 (Tie2) pathway also plays a critical role in angiogenesis and vascular stability. Additionally, newly developed treatment delivery systems have been designed to greatly reduce the frequency of injections. In targeting the Tie2 pathway and utilizing a sustained release delivery system, patients may experience improved visual outcomes and a reduced burden of treatment.

## 1. Introduction

With a predicted worldwide prevalence of 288 million adults by 2040, age-related macular degeneration (AMD) is one of the leading causes of vision loss among individuals over 50 years old [1,2]. Recent advances in anti-vascular endothelial growth factor (anti-VEGF) agents have revolutionized the management of neovascular (wet) AMD, allowing patients to improve short-term visual acuity while slowing the progression of vision loss in the long-term. Despite the tremendous success of anti-VEGF therapy, patients are experiencing an increased burden of treatment due to frequent injections and multiple office visits to assess treatment response. This can be demanding for patients and ultimately result in reduced patient adherence and an inconsistent level of treatment [3]. Additionally, numerous retrospective studies have reported sub-optimal responder rates for anti-VEGF agents to be as high as 10–15% [4,5]. It has been hypothesized that this high rate of sub-responders is largely due to angiogenic factors and pathways other than VEGF playing a role in the disease progression. Long-term studies evaluating the efficacy of anti-VEGF agents have demonstrated that initial improvements in vision over the first two years are not always sustained over longer periods of time [6]. This may be the result of under treatment in the real world setting or potentially the development of macular atrophy in these patients.

Emerging treatment modalities for neovascular AMD aim to overcome the shortcomings of current agents by providing a more predictable improvement of vision while reducing the frequency of injections that are needed. Among these modalities are emerging agents that target alternative angiogenic pathways, and delivery systems that allow a constant and sustained level of treatment.

## 2. The Tie2 Pathway

Located on vascular endothelial cells, tyrosine kinase with immunoglobulin-like and epidermal growth factor-like domains 2 (Tie2) is a trans membrane receptor that serves as a binding site for the angiopoietin family of ligands that includes angiopoietin-1 (Ang1) and angiopoietin-2 (Ang2). Ang1 is an endogenous full agonist of Tie2 that is produced in pericytes surrounding the retinal vasculature. Once bound to Tie2, Ang1 functions to phosphorylate the receptor and, thereby, activate downstream pathways that ultimately suppress vascular permeability and maintain vascular stability [7]. Conversely, Ang2 is an endogenous weak, partial agonist of Tie2 that competes with Ang1 to suppress phosphorylation and the activation of Tie2 [8,9]. Preclinical studies have shown that embryonic mice that are deficient in the Tie2 receptor die by embryonic day 10.5 due to incorrect vessel organization and failure of vessels to mature [10,11]. Additionally, mice deficient in Ang1 result in a phenotype that is similar to that of Tie2-deficient mice, while overexpression of Ang2 in mice results in a phenotype reminiscent of the Tie2 and Ang1-deficient mice, demonstrating the opposing activities of Ang1 and Ang2 [12].

The interaction between the angiopoietin family and VEGF has been studied in the ocular setting using mice that co-express VEGF-A and ANG-1. When expressed simultaneously with VEGF-A, Ang1 prevented retinal detachment and blocked neovascularization [13]. Despite Ang1 effectively suppressing neovascularization, there was no observed impact on lesions that were previously established [13]. Additionally, early studies using mice have demonstrated that despite increased expression of VEGF in the inner surface of the retina, neovascularization and vascular leakage do not occur unless in the presence of elevated levels of Ang2 [14,15,16,17]. Ang2 has also been further studied for its role in upregulating inflammation via a mechanism of inducing endothelial cells to become more sensitive to the effect of tumor necrosis factor-alpha (TNFα) [18]. In fact, Ang2 is believed to be stored within endothelial cells in Weibel–Palade bodies where it is rapidly released under the influence of angiogenic, inflammatory, and other cytokines [18,19]. Further significance of Ang2 is established from studies that have shown elevated levels of Ang2 and VEGF-A in the vitreous of diabetic patients undergoing a vitrectomy [20]. The levels of Ang2 and VEGF-A were shown to correlate with each other as well as disease severity [20,21]. Thus, Ang2 appears to play a considerable role in both angiogenesis and inflammation, and has become an attractive target for therapeutics aimed at inhibiting angiogenesis [22].

## 3. Emerging Therapeutics Targeting the Tie2 Pathway

RG7716 (Roche/Genentech) is a bispecific monoclonal antibody that has been designed to bind VEGF-A with one arm and Ang2 with the other arm. Through this mechanism, RG7716 has the ability to target multiple pathways implicated in angiogenesis.

This antibody is currently being studied as an intravitreal therapy in the treatment of neovascular age-related macular degeneration. A phase I clinical study has demonstrated that the antibody is safe and well tolerated, with no unexpected adverse events [23]. This study consisted of two treatment groups: a single ascending dose group and a multiple ascending dose group. In the single ascending dose group, patients received 0.5 mg, 1.5 mg, 3 mg or 6 mg with a study duration of up to 16 weeks. In the multiple ascending group, patient received either 3 mg or 6 mg of the drug with a total of three administrations in monthly intervals for a study duration of 24 weeks. The single ascending dose group documented a median improvement of best-corrected visual acuity of 7 letters, while the multiple ascending group demonstrated a median improvement in visual acuity of 7.5 letters [24,25]. Two phase II studies are underway to assess the efficacy of RG7716 in treating neovascular AMD, including a 76-patient extended-dosing study (STAIRWAY) and a 273-patient randomized, double-masked study (AVENUE) [26,27]. STAIRWAY and AVENUE have an estimated completion time of early 2018 with the data becoming available in the middle of 2018 [26,27]. Together, these studies will provide further clarity into the efficacy and durability of RG7716 in the management of neovascular AMD.

## 4. Sustained Release: Ranibizumab Port Delivery System

The ranibizumab port delivery system is a refillable implant that is placed beneath the conjunctiva and is designed to provide sustained release of ranibizumab into the vitreous [28,29]. The port delivery system has the potential to greatly reduce the burden of frequent injections. For this reason, the ranibizumab port delivery system may provide physicians with an improved tool in the management of neovascular AMD. In a phase I study, the port delivery system was shown to be well tolerated and demonstrated an improvement in best-corrected visual acuity (BCVA) in treatment-naïve patients with neovascular age-related macular degeneration [28,29]. The ranibizumab port delivery system is currently being evaluated in a randomized, double blind phase II study (LADDER) with a 220 patient enrollment [30]. LADDER will assess the safety and efficacy of ranibizumab delivered via the port delivery system compared to the standard of care intravitreal injections of ranibizumab.

## 5. Conclusions

Anti-VEGF therapies will likely remain the treatment of choice for neovascular AMD for many years to come; however, emerging therapies that target alternative pathways and the development of new delivery systems show great promise for improving the frequency of injections and visual outcomes. Early clinical studies have shown that targeting the Tie2 pathway and utilizing a sustained release delivery system are both potential therapeutic strategies as we advance beyond traditional anti-VEGF intravitreal injection modalities. The data from ongoing large clinical trials to assess efficacy and safety will show if these treatments will become viable options to treat patients with neovascular AMD in the future.
